# Supplementary material for: Hybrid Models and Biological Model Reduction with PyDSTool
Source: PLoS Comput Biol. 2012 Aug 9;8(8):e1002628. doi: 10.1371/journal.pcbi.1002628 (PMC3415397; doi:10.1371/journal.pcbi.1002628)
Supplement: Text S4 — Complete source code for the PyDSTool package (version 0.88.120504). Includes API documentation and help files linking to web pages. This file is identical to the current public release on Sourceforge.net. (ZIP) [file pcbi.1002628.s004.zip › PyDSTool/html/PyDSTool.Toolbox.optimizers.line_search.scaled_line_search-pysrc.html]

xml version="1.0" encoding="ascii"?


PyDSTool.Toolbox.optimizers.line\_search.scaled\_line\_search


| Home | Trees | Indices | Help | PyDSTool |
| --- | --- | --- | --- | --- |

Package PyDSTool ::
Package Toolbox ::
Package optimizers ::
Package line\_search ::
Module scaled\_line\_search

[hide private]

[frames] | no frames]

# Source Code for Module PyDSTool.Toolbox.optimizers.line\_search.scaled\_line\_search

```
  1  import numpy as np 
  2   
  3   


4 -class ScaledLineSearch(object):


5      """ 
  6      A simple line search, takes a point, adds a step and returns it 
  7      Scales step according to given scales of the parameters and ignores 
  8      *magnitude* of gradient. 
  9   
 10      (in early development and experimental only at this point) 
 11      """ 


12 -    def __init__(self, max_step=1, step_mod=3, **kwargs):


13          """ 
 14          Needs to have : 
 15          - nothing 
 16          Can have : 
 17          - max_step: a maximum step control, a scalar or vector to restrict step size 
 18          in each direction (default 1) 
 19          - step_mod: a factor to divide the step when back-tracking (default 3) 
 20          - max_reduce_fac: max_step divided by this is the smallest step that will be tried (default 2000), 
 21          """ 
 22          self.maxStepSize = max_step 
 23          self.stepMod = step_mod 
 24          if np.isscalar(max_step): 
 25              self.basis = None 
 26              self.dim = None 
 27          else: 
 28              self.dim = len(max_step) 
 29              self.basis = np.identity(self.dim) 
 30          try: 
 31              self.filter = kwargs['filter'] 
 32          except KeyError: 
 33              self.filter = False 
 34          try: 
 35              self.maxReduceFac = kwargs['max_reduce_fac'] 
 36          except KeyError: 
 37              self.maxReduceFac = 7 #5000

 38  #        try: 
 39  #            self.use_dirs = kwargs['use_directions'] 
 40  #        except KeyError: 
 41  #            self.use_dirs = np.zeros((self.dim,),float) 
 42   
 43   


44 -    def __call__(self, origin, state, **kwargs):


45          """ 
 46          Returns a good candidate 
 47          Parameters : 
 48          - origin is the origin of the search 
 49          - state is the state of the optimizer 
 50          """ 
 51          fun = kwargs['function'] 
 52          d = state['direction']/np.linalg.norm(state['direction']) 
 53          # filter directions that are too large 
 54          if self.filter: 
 55              ndabs_log = -np.log10(np.abs(d)) 
 56              mean_log = np.mean(ndabs_log) 
 57              #print "\n ** MEAN =", mean_log 
 58              direction = (ndabs_log > mean_log-1.5).astype(int)*d 
 59          else: 
 60              direction = d 
 61          state['direction'] = direction 
 62  ##        for pos, d in enumerate(direction): 
 63  ##            use_dir = self.use_dirs[pos] 
 64  ##            if use_dir * d < 0: 
 65  ##                # directions don't match so don't move in this direction 
 66  ##                direction[pos] = 0 
 67          maxStepSize = self.maxStepSize 
 68          if np.isscalar(maxStepSize): 
 69              stepSize = maxStepSize 
 70          else: 
 71              stepfacs = np.zeros(self.dim) 
 72              for d in range(self.dim): 
 73                  # explicit loop so as to catch any ZeroDivisionErrors 
 74                  try: 
 75                      stepfacs[d] = abs(maxStepSize[d] / direction[d]) 
 76                  except ZeroDivisionError: 
 77                      # Direction is orthogonal to this parameter direction, 
 78                      # so ensure won't choose this as the minimum step size 
 79                      stepfacs[d] = Inf 
 80              # Stop stepping with giant sizes if direction vector has strong 
 81              # separation of scales 
 82              stepSize = min(stepfacs) 
 83  #            print "direction = ", direction 
 84  #            print "step = ", step 
 85          i = 1 
 86          old_value = state['old_value'] 
 87          not_done = True 
 88  #        print "** TEMP: Hardwiring step size to be 0.0005" 
 89  #        stepSize = 0.0005 
 90          init_step = stepSize 
 91          while not_done: 
 92              print "\nLinestep: i =", i, "step size =", stepSize, "direction =\n", direction 
 93              p = origin + i * stepSize * direction 
 94              print "Testing p = ", p 
 95              new_value = fun(p) 
 96              if new_value < old_value: 
 97                  i += 1 
 98                  old_value = new_value 
 99              else: 
100                  if i == 1: 
101                      # don't shrink step size to be less than 1/maxReduceFac of initial 
102                      if stepSize*self.maxReduceFac < init_step: 
103                          not_done = False 
104                          p = origin + (i-1) * stepSize * direction 
105                      else: 
106                          stepSize /= self.stepMod 
107                  else: 
108                      # had found a working step but it's no longer stepping to lower residuals 
109                      not_done = False 
110                      p = origin + (i-1) * stepSize * direction 
111          state['alpha_step'] = stepSize 
112          return p

113
```

  


| Home | Trees | Indices | Help | PyDSTool |
| --- | --- | --- | --- | --- |

|  |  |
| --- | --- |
| Generated by Epydoc 3.0.1 on Fri May 4 15:24:22 2012 | http://epydoc.sourceforge.net |
